# Epicardial adipose tissue thickness and growth differentiation factor 15 in axial spondyloarthritis: A cross-sectional study

**DOI:** 10.15537/smj.2022.43.9.20220304

**Published:** 2022-09

**Authors:** Mehmet Okçu, Fatmanur A. Koçak, Senem Şaş, Kenan Güçlü

**Affiliations:** *From the Department of Physical Medicine and Rehabilitation (Okçu), Marmara University Faculty of Medicine, Istanbul; from the Department of Physical Medicine and Rehabilitation (Koçak), Kırsehir Ahi Evran University Faculty of Medicine; from the Department of Physical Medicine and Rehabilitation (Şaş), Division of Rheumatology, Kayseri, Erciyes University Faculty of Medicine; and from the Department of Biochemistry (Güçlü), Kırsehir Ahi Evran University Faculty of Medicine, Kırsehir, Turkey.*

**Keywords:** axial spondylarthritis, growth differentiation factor 15, epicardial adipose tissue, ankylosing spondylitis

## Abstract

**Objectives:**

To investigate growth differentiation factor-15 (GDF-15) levels and the thickness of epicardial adipose tissue (EAT) in patients with axial spondyloarthritis (axSpA) and to evaluate their relationship with functional status, disease activity, disease duration, and the type of medical treatment received by the patients.

**Methods:**

This cross-sectional study was carried out at Kırşehir Ahievran University School of Medicine between February and June 2020. Twenty-nine healthy controls and 44 patients with axSpA were included in the study. Gender, age, erythrocyte sedimentation rate, GDF-15, body mass index, complete blood count, ejection fraction, the EAT thickness, and C-reactive protein of all participants were recorded. Ankylosing Spondylitis Quality of Life Index, Bath Ankylosing Spondylitis Functional Index, the disease duration, Bath Ankylosing Spondylitis Metrology Index, and Bath Ankylosing Spondylitis Disease Activity Index scores of patients with axSpA were noted.

**Results:**

Epicardial adipose tissue thickness values (0.35±0.09 cm) in the AxSpA group were higher compared to the control group (0.26±0.06 cm) (*p*<0.01). Growth differentiation factor-15 levels of the control group and axSpA group were similar. The treatment received by the patients did not have a significant relationship with EAT thickness and GDF-15. Bath Ankylosing Spondylitis Functional Index scores, disease duration, and age were significantly positively correlated with GDF-15 levels.

**Conclusion:**

In this study, EAT thickness values were found to be significantly higher in the axSpA group. In addition, GDF-15 was positively correlated with age, Bath Ankylosing Spondylitis Functional Index score, and disease duration.

Axial spondylarthritis (axSpA) is a disease in which there is an inflammation and pain in the axial joints and spinal region.^1^ Spinal limitation, uveitis, and inflammatory bowel disease may accompany axSpA. ^2^ It has been reported that 10-30% of patients with AxSpA have cardiac involvement. The incidence of cardiac involvement is higher in patients who are HLA-B27 positive.^3,4^ Mortality rates due to cardiac problems are higher in patients with axSpA compared to the normal population.^4,5^ Some authors suggested that this increase in mortality was due to increased inflammation in axSpA. However, the reason for the increased mortality due to cardiovascular diseases (CVD) in axSpA has not been fully elucidated.^4-6^

Atherosclerosis ranks first among the reasons for coronary artery disease (CAD).^7^ Epicardial adipose tissue (EAT), which also has systemic effects, is localized in the upper part of the left ventricle. It has been reported that EAT thickness is associated with the severity of CAD.^8^ In addition, biopsy studies have shown that EAT contains large amounts of mediators such as interleukin-1 (IL-1), Micro RNA, and IL -6. ^4,9^ The EAT of patients with rheumatoid arthritis was found to be thicker than that of healthy individuals.^10, 11^

Growth differentiation factor-15 (GDF-15) affects cell growth and differentiation and is secreted by activated macrophages.^12^ For GDF-15 to be secreted from resting macrophages, these macrophages must be stimulated by mediators such as TNF, IL-1, and macrophage colony stimulating factor.^13^ Growth differentiation factor-15 is also known as placental transforming growth factor beta (PTGFB), PLAB, Non-steroidal anti-inflammatory drugs activated gene-1 (NAG-1), prostate derived factor (PDF), macrophage inhibitory cytokine-1 (MIC-1).^14^ Growth differentiation factor-15 has been found to be upregulated in atherosclerosis and related with increased cardiovascular risk.^13,15^ In addition, studies are reporting that the GDF-15 levels are increased in rheumatic diseases.^12,16^ In order to reduce mortality and morbidity more effectively, it will be useful to demonstrate the presence of atherosclerosis with easy, inexpensive, noninvasive tests before cardiovascular symptoms begin. Thus, in patients who are determined to be at high risk, treatment can be started earlier and complications can be prevented.

Studies on EAT thickness and GDF-15 in patients with axSpA are very limited in the literature. This study aimed to examine EAT thickness and GDF-15 levels in axSpA. In addition, to investigate the relationship of GDF-15 levels and EAT thickness with parameters such as disease duration, functional status, the severity of disease, and the type of medical treatment received by the patients.

## Methods

This study was carried out after approval by the Kırşehir Ahievran University School of Medicine Clinical Research Ethics Committee (Date: 11.02.2020, No: 2020-02/12). Patients were included in the study after informed consent was obtained. The study was carried out in accordance with the Declaration of Helsinki Ethical Principles. This cross-sectional study was carried out at Kırşehir Ahievran University School of Medicine between February 2020 and June 2020.

Inclusion criteria were aged 18-65 years and to have a diagnosis of axSpA according to the classification criteria of the Assessment of SpondyloArthritis International Society (ASAS).^17^ Patients were selected consecutively from the patients who visited the Kirsehir Ahi Evran University School of Medicine Hospital Physical Medicine and Rehabilitation outpatient clinic. Exclusion criteria were as follows: patients with a history of hypertension, connective tissue disease, renal and hepatic failure, diabetes mellitus, cerebrovascular disease, myocardial infarction, cardiovascular disease, chronic steroid use, immunosuppressive therapy, retinoid, lipid-lowering therapy, antiplatelet therapy, antihypertensive therapy, and cancer history. As the control group, healthy volunteers aged 18-65 years, who did not have the diseases in the above-mentioned exclusion criteria and did not have axSpA diagnosis, were taken consecutively.

The age, gender, smoking, weight, and height of the participants were noted. Bath Ankylosing Spondylitis Metrology Index (BASMI), Bath Ankylosing Spondylitis Disease Activity Index (BASDAI), chest expansion value, disease duration, Ankylosing Spondylitis Quality of Life Index (ASQoL) scores, Bath Ankylosing Spondylitis Functional Index (BASFI) scores, enthesitis count, erythrocyte sedimentation rates (ESR), complete blood count (CBC), and C-reactive protein (CRP) levels were recorded.

Bath Ankylosing Spondylitis Disease Activity Index evaluates disease activity in axSpA. Patients are scored from 0 to 10. Lower scores mean the disease is less active.^18^ A BASDAI score of ≥4 indicates high disease activity, <2 indicates low disease activity, and ≥2 to <4 indicates moderate disease activity.^19,20^ Bath Ankylosing Spondylitis Functional Index evaluates the functional level in ankylosing spondylitis (AS). Higher values indicate worse functional status.^21^ ASQoL is a scale consisting of 18 questions. Patients are scored from 0 to 18. Ankylosing Spondylitis Quality of Life Index evaluates the quality of life in AS. Patients with low quality of life score higher.^22^ Bath Ankylosing Spondylitis Metrology Index evaluates joint mobility at various sites in patients with AS. It is based on lumbar lateral flexion, cervical rotation, tragus-wall distance, malleolar distance, and modified Schober measurements. In each measurement, the patient gets values from 0 to 10. The arithmetic mean of these values is the BASMI value.^23^ The Turkish version of these scales was translated and validated.^24-26^ An experienced cardiologist who did not know the diagnosis of the participants made echocardiographic evaluations of the patients. Echocardiographic evaluations of the patients were performed in the left lateral decubitus position. Measurements were made using transthoracic echocardiography (Vivid S5, GE Vingmed Ultrasound AS, Horten, Norway). Epicardial adipose tissue was determined as echo-free space in the pericardial layers and its thickness was measured perpendicular to the free wall of the right ventricle at the end of systole. Two echocardiographic windows, parasternal long axis and short axis, were used. For parasternal short axis view, epicardial fat tissue thickness was measured 2 cm from the ventricular septum in the right ventricular free wall. The mean value of 3 cardiac cycles was calculated and recorded for each echocardiographic window. Ejection fraction (EF) and EAT thickness were recorded.

Venous blood samples were transferred to K2EDTA-containing tubes and routine biochemistry tubes. Before centrifugation, the sample in the biochemistry tubes was left for 30 minutes to coagulate. The samples were then centrifuged (10 minutes [mins], 3000 rpm). The obtained serums were collected in aliquots at ^-^80°C before performing the GDF-15 analysis. Serums were thawed at room temperature before analysis. C-reactive protein, CBC, and ESR were measured immediately. Measurement of ESR levels was made with an auto-analyzer using the modified Westergren sedimentation technique (Siena, Italy, Diesse Diagnostica Senese Spa, Ves-Matic Cube 200). Measurement of CRP levels was performed with a routine biochemistry auto-analyzer (Roche Diagnostic Corporation, Cobas 8000, Mannheim, Germany). Measurement of CBC was performed on an automated analyzer (Sysmex Company, Sysmex XN-1000, Japan). Measurement of serum GDF-15 levels was performed by an ELISA (Relassay, Turkey, Gaziantep). The inter CVs for GDF-15 was <12 and intraassay CVs for GDF-15 was <10%. The limit of detection (LOD) of the assay was 5.09 ng/L.

### Statistical analysis

Statistical analyzes of the study were performed with the Statistical Package for the Social Sciences, version 21.0 for Windows (IBM Corp., USA). According to the normality assumption, the explanatory statistics of the variables are summarized as mean±standard deviation, median (25th percentile -75th percentile), and frequencies (n) (%). Normality assumption was tested using Shapiro-Wilk tests and Kolmogorov-Smirnov. Group comparisons were made using analysis of variance (ANOVA), independent t-test, the Kruskal-Wallis test, and the Mann-Whitney U tests. Multiple comparisons of groups with significant differences were made using the Mann-Whitney U test and Duncan’s multiple comparison test. Pairwise comparisons of groups with significant differences between them as a result of the Kruskal-Wallis test were made using the Mann-Whitney U test and evaluated by applying Bonferroni correction (0.05/group number). The comparison of the groups with significant differences between them as a result of ANOVA was performed with the DUNCAN multiple comparison test. Spearman’s Rho correlation analysis was used to test the relationships between continuous variables that did not satisfy the assumption of normality.

## Results

The study was carried out with 29 healthy controls and 44 axSpA patients. The mean age of the control group and axSpA group was similar. The distribution of the genders between the axSpA and control groups was homogeneous. White blood cell (WBC), body mass index (BMI), hemoglobin, platelet, ESR, CRP, and EF values of the control and axSpA groups were statistically similar. Epicardial adipose tissue thickness values of axSpA patients were higher. (Table 1)

**Table 1:** - Comparisons of clinical and demographic parameters of axSpA and control group

Characteristics	axSpA, n=44	Control, n=29	*P*-value
Age (years)	38.65±10.04	41.48±11.72	0.998
***Gender, n (%)***			
Female	13 (61.9%)	8 (38.1%)	0.856
Male	31 (59.6%)	21 (40.4%)	
BMI (kg/m^2^)	25.79±4.16	25.89±2.89	0.911
GDF-15 (ng/L)	103.11 (48.40-166.56)	86.37 (37.45-157.51)	0.543
WBC (10^3^/ µL)	8332.27±1468.12	8113.44±1729.96	0.564
ESR (mm/h)	10 (4.0-18.75)	7 (4.0-17.0)	0.568
Hemoglobin(g/dL)	14.30±1.36	14.65±1.36	0.295
CRP (mg/dL)	0.55 (0.14-1.05)	0.33 (0.13-0.77)	0.245
Platelet count(10^3^/µL)	287479.5422±58592	269975.86±51697.28	0.195
EF (%)	63.45±2.52	63.89±2.71	0.480
EAT thickness (cm)	0.35±0.09	0.26±0.06	<0.001

AxSpA: axial spondyloarthritis, ESR: erythrocyte sedimentation rate, BMI: Body mass index, GDF-15: growth differentiation factor 15, CRP: C-reactive protein; EF: ejection fraction, EAT: epicardial adipose tissue; cm: centimeter, WBC: white blood cell

Growth differentiation factor-15 was positively correlated with BASFI, disease duration, and age. The relationships between GDF-15 and EAT thickness variables with each other and with other variables are summarized in (Table 2).

**Table 2:** - Relationships of demographic and clinical parameters of patients in the AxSpA group with GDF-15 level and epicardial adipose tissue thickness values (N=44).

Characteristics	GDF-15	EAT thickness
GDF-15	1	0.196
Age (years)	0.318**	0.050
BMI (kg/m^2^)	-0.056	-0.085
Disease duration (years)	0.345*	0.072
WBC (10^3^/ µL)	0.103	-0.96
ESR (mm/h)	0.127	0.082
Hemoglobin(g/dL)	0.015	0.039
CRP (mg/dL)	-0.040	0.182
Platelet count(10^3^/µL)	0.112	0.059
Smoking duration	0.091	-0.126
Chest expansion	-0.101	-0.191
BASDAI	0.131	0.182
BASFI	0.334*	0.084
BASMI	0.135	0.004
ASQoL	0.155	-0.073
EF (%)	0.153	-0.026
EAT thickness	0.196	1

AxSpA: axial spondyloarthritis; BMI: body mass index, ASQoL: Ankylosing Spondylitis Quality of Life questionnaire, GDF-15: growth differentiation factor 15, BASMI: Bath Ankylosing Spondylitis Metrology Index, WBC: white blood cell, BASFI: Bath Ankylosing Spondylitis Functional Index, EF: ejection fraction, EAT: epicardial adipose tissue, BASDAI: Bath Ankylosing Spondylitis Disease Activity Index; ESR, erythrocyte sedimentation rate, CRP: c-reactive protein

Patients with axSpA were divided into 2 groups as those who received biological disease-modifying antirheumatic drugs (BD) (BD group n=22) and those who did not (non-BD group; n=22) (Table 3).

**Table 3:** - Effects of different groups on growth differentiation factor-15 (GDF-15), EF, and thickness of EAT.

Groups	GDF-15	EF	EAT thickness (cm)
BD group (n=22)	103.11 (65.29-167.86)	63.86±2.64	0.36±0.10
Non-BD group (n=22)	95.42 (43.26-164.85)	63.04±2.39	0.35±0.09
Control (n=29)	86.37 (37.45-157.51)	63.89±2.71	0.26.06
P	0.543	0.480	<0.001
P^(BD-non BD)^	0.467	0.288	0.972
P^(BD-C)^	0.382	0.966	<0.001
P^(Non BD -C)^	0.879	0.250	<0.001

BD: biological disease-modifying antirheumatic drugs, C: controC: control, EF: ejection fraction, EAT: epicardial adipose tissue, cm: centimeter, P: *p*-**value**

The relationship between gender, smoking status, and the number of enthesitis with GDF-15 and EAT thickness values were not statistically significant (Table 4).

**Table 4:** - Relations of gender, smoking status and number of enthesitis with growth differentiation factor-15 (GDF-15) and EAT thickness of patients in the AxSpA group.

Factors	GDF-15	*P*-value	EAT thickness	*P*-value
***Gender***				
Male (n=31)	99.18 (48.40-160.52)	0.951	0.33 (0.24-0.39)	0.207
Female (n=13)	115.16 (37.79-192.40)		0.26 (0.22-0.36)	
***Smoking***				
Smoker (n=25)	85.18 (46.11-168.50)	0.558	0.36 (0.30-0.42)	0.577
Non-smoker (n=19)	115.16 (59.61-159.95)		0.34 (0.23-0.43)	
***Number of enthesitis***				
0 (n=33)	115.16 (57.41-165.49)	0.663	0.35 (0.24-0.43)	0.801
1 (n=4)	94.84 (62.72-160.54)		0.37 (0.33-0.45)	
2 (n=7)	46.13 (36.35-235.14)		0.36 (0.33-0.42)	
***Disease activity***				
Patients with HDA (BASDAI≥4) (n=25)	141.52±114.31	0.241	0.37±0.07	0.184
Patients without HDA (BASDAI<4) (n=19)	95.06±54.36		0.34±0.12	

AxSpA: axial spondyloarthritis, EAT: epicardial adipose tissue, HDA: high disease activity, EF: ejection fraction

## Discussion

In this study, EAT thickness values of axSpA patients were higher than healthy controls. Growth differentiation factor-15 was found to be correlated with disease duration, age, and BASFI.

Cardiovascular involvement causes increased mortality and morbidity in patients with AxSpA. Therefore, cardiac involvement should be detected as early as possible in the early stages of the process.^4,5,27^ It is not fully clear why the cardiac risk is increased in patients with axSpA, but some researchers blame increased inflammation for this increased cardiac risk. In patients with axSpA, chronic inflammation contributes significantly to atherosclerosis formation steps such as early plaque instability, thrombus formation, and atheroma formation.^28^ In addition to risk factors for classic CVD, oxidative stress, endothelial dysfunction, direct endothelial damage caused by inflammation, and changes in lipoprotein concentrations were also found to be effective in accelerated atherosclerosis in rheumatic disease.^29^ It is recommended by The European League Against Rheumatism that more aggressive screening and treatment for CVD should be performed in people with RA because of the higher risk of CVD.^28^ Cardiac death rates are higher in patients with axSpA than in the normal population. Mortality in patients with axSpA is positively associated with proinflammatory cytokines.^4^ However, the pathophysiology of the increased cardiovascular risk in axSpA has not been fully elucidated. There remains a need for noninvasive methods to help early recognition of cardiovascular risk in patients with axSpA to start treatment earlier.

Epicardial adipose tissue is associated with CAD, it is an adipose tissue surrounding the heart, especially the subepicardial coronary vessels.^9,30^ Under physiological conditions, EAT has been claimed to buffer toxic fatty acids between the local vascular beds and the myocardium.^31^ Epicardial adipose tissue has been shown to express numerous genes for atherosclerosis-related cytokines and proteins.^32,33^ It has been shown that echocardiographic evaluation of EAT thickness can be an inexpensive and noninvasive assessment tool for determining CAD risk.^9^ Studies show that patients with rheumatoid arthritis have a thicker EAT than healthy individuals.^10,11^ However, there are very few studies investigating the relationship between axSpA and EAT thickness.^4,28^ Growth differentiation factor-15 is associated with atherosclerosis and cardiac risk.^13-15^ Some studies that found GDF-15 levels to be higher in RA and axSpA than in healthy people.^12,16^ However, data on this subject are insufficient.

Similar to the current study, Boyraz et al^4^ and Sürücü et al,^28^ found that patients with AS had a thicker EAT than healthy controls. Song et al^16^ found that the GDF-15 levels of the SpA group were higher compared to the GDF-15 levels of the healthy controls. In addition, they found that GDF-15 was associated with CRP levels and bone erosion in magnetic resonance imaging.^16^ In the current study, a significant correlation was found between GDF-15 levels, functional status, and disease duration, but GDF-15 levels of axSpA patients and the control group were similar. This may be due to the difference in parameters such as the number of participants in the study, disease activity, and disease duration. In addition, it was difficult to find participants in the study due to the COVID pandemic that emerged in the country shortly after the start of the study. Therefore, the number of participants in this study was small. The low number of participants reduces the power of the study. The lack of statistically significant difference in the GDF-15 level may be due to the small sample size and low power. However, the strength of this study is that it is one of the few studies examining GDF 15 and EAT thickness in axSpA.

Lambrecht et al^34^ found that serum GDF-15 levels of patients with SpA were significantly lower than those of patients with RA. They determined that the synovial fluid of participants with SpA had higher GDF-15 levels than serum, and reported that GDF-15 in synovial fluid and serum were similar in patients with RA. They also found that there was no relationship between CRP, disease activity, and GDF-15 level.^34^ Similar to Lambrecht et al’s^34^ findings, the association between GDF-15 and CRP was also statistically insignificant in the current study. In this study, BASDAI was not correlated with GDF-15; however, a correlation was found between BASFI and GDF-15. Robertson et al^35^ followed patients with AS for 5 years and reported that BASDAI was more stable and BASFI worsened over time. Bath Ankylosing Spondylitis Disease Activity Index assesses fatigue, localized tenderness in the dorsal region, morning stiffness, and spinal and peripheral joint pain, however, BASFI evaluates daily activities and the ability to achieve responsibilities in daily life. Bath Ankylosing Spondylitis Functional Index has a stronger correlation with disease duration than BASDAI.Therefore, the correlation between GDF-15 and BASFI in the current study may be related to the correlation between GDF-15 and disease duration.

### Study limitations

Although the present study was performed in patients with axial rather than peripheral SpA, the lack of synovial evaluation can be considered a limitation of this study. The lack of radiologic evaluation and further cardiologic evaluations can also be counted as limitations of our study. The small sample size is one of the most important limitations of this study. The lack of statistically significant difference in some parameters may be due to the small sample size. However, despite all these limitations, the strength of this study is that it is one of the few studies examining GDF 15 and EAT thickness in axSpA.

In conclusion, patients with axSpA have significantly higher EAT levels than controls. GDF-15 levels were positively correlated with BASFI scores, disease duration, and age. Prospective, further, and larger studies should be designed to reveal the associations more clearly.

## References

[1] Resende GG , Meirelles EdS , Marques CDL , Chiereghin A , Lyrio AM , Ximenes AC , et al. The Brazilian Society of Rheumatology guidelines for axial spondyloarthritis – 2019. Adv Rheumatol 2020; 60: 19. 32171329 10.1186/s42358-020-0116-2

[2] Carli L , Calabresi E , Governato G , Braun J. One year in review 2018: axial spondyloarthritis. Clin Exp Rheumatol 2019; 37: 889–898. 31796161

[3] Baniaamam M , Heslinga SC , Konings TC , Handoko ML , Kamp O , van Halm VP , et al. Aortic root diameter is associated with HLA-B27: identifying the patient with ankylosing spondylitis at risk for aortic valve regurgitation. Rheumatol Int 2022; 42: 683–688. 34729637 10.1007/s00296-021-05040-w PMC8940876

[4] Boyraz I , Onur Caglar S , Erdem F , Yazici M , Yazici S , Koc B , et al. Assessment of relation between neutrophil lympocyte, platelet lympocyte ratios and epicardial fat thickness in patients with ankylosing spondylitis. Med Glas (Zenica) 2016; 13: 14–17. 26827703 10.17392/832-16

[5] Peters MJ , Symmons DP , McCarey D , Dijkmans BA , Nicola P , Kvien TK , et al. EULAR evidence-based recommendations for cardiovascular risk management in patients with rheumatoid arthritis and other forms of inflammatory arthritis. Ann Rheum Dis 2010; 69: 325–331. 19773290 10.1136/ard.2009.113696

[6] Papagoras C , Voulgari PV , Drosos AA. Atherosclerosis and cardiovascular disease in the spondyloarthritides, particularly ankylosing spondylitis and psoriatic arthritis. Clin Exp Rheumatol 2013; 31: 612–620. 23406817

[7] Al-Ghamdi SH , Aldosari KH , AlAjmi MM. Patterns and determinants of treatment for coronary artery disease. A cross-sectional study in the Kingdom of Saudi Arabia. Saudi Med J 2021; 42: 895–902. 34344814 10.15537/smj.2021.42.8.20210219 PMC9195552

[8] Yun KH , Rhee SJ , Yoo NJ , Oh SK , Kim N-H , Jeong J-W , et al. Relationship between the echocardiographic epicardial adipose tissue thickness and serum adiponectin in patients with angina. J Cardiovasc Ultrasound 2009; 17: 121–126. 20661336 10.4250/jcu.2009.17.4.121 PMC2889395

[9] Jeong JW , Jeong MH , Yun KH , Oh SK , Park EM , Kim YK , et al. Echocardiographic epicardial fat thickness and coronary artery disease. Circ J 2007; 71: 9. 17384455 10.1253/circj.71.536

[10] Fatma E , Bunyamin K , Savas S , Mehmet U , Selma Y , Ismail B , et al. Epicardial fat thickness in patients with rheumatoid arthritis. Afr Health Sci 2015; 15: 489–495. 26124795 10.4314/ahs.v15i2.23 PMC4480478

[11] Lima-Martínez MM , Campo E , Salazar J , Paoli M , Maldonado I , Acosta C , et al. Epicardial fat thickness as cardiovascular risk factor and therapeutic target in patients with rheumatoid arthritis treated with biological and nonbiological therapies. Arthritis 2014; 2014: 782850. 25574390 10.1155/2014/782850 PMC4276696

[12] Tanrıkulu O , Sarıyıldız MA , Batmaz İ , Yazmalar L , Polat N , Kaplan İ , et al. Serum GDF-15 level in rheumatoid arthritis: relationship with disease activity and subclinical atherosclerosis. Acta Méd Port 2017; 42: 66–72. 27679935

[13] Brown DA , Breit SN , Buring J , Fairlie WD , Bauskin AR , Liu T , et al. Concentration in plasma of macrophage inhibitory cytokine-1 and risk of cardiovascular events in women: a nested case-control study. Lancet 2002; 359: 2159–2163. 12090982 10.1016/S0140-6736(02)09093-1

[14] Guo TM , Yan Y , Cao WN , Liu Q , Zhu HY , Yang L , et al. Predictive value of microRNA-132 and its target gene NAG-1 in evaluating therapeutic efficacy of non-steroidal anti-inflammatory drugs treatment in patients with ankylosing spondylitis. Clin Rheumatol 2018; 37: 1281–1293. 29497899 10.1007/s10067-018-4017-2

[15] Ago T , Sadoshima J. GDF15, a cardioprotective TGF-beta superfamily protein. Circulation research 2006; 98: 294–297. 16484622 10.1161/01.RES.0000207919.83894.9d

[16] Song Y , Cui Y , Zhang X , Lin H , Zhang G , Zeng H , et al. Increased serum levels of MIC1/GDF15 correlated with bone erosion in spondyloarthritis: A pilot study. Medicine (Baltimore) 2018; 97: e13733. 30572513 10.1097/MD.0000000000013733 PMC6320148

[17] Rudwaleit M , van der Heijde D , Landewé R , Listing J , Akkoc N , Brandt J , et al. The development of Assessment of SpondyloArthritis international Society classification criteria for axial spondyloarthritis (part II): validation and final selection. Ann Rheum Dis 2009; 68: 777–783. 19297344 10.1136/ard.2009.108233

[18] Calin A , Nakache JP , Gueguen A , Zeidler H , Mielants H , Dougados M. Defining disease activity in ankylosing spondylitis: is a combination of variables (Bath Ankylosing Spondylitis Disease Activity Index) an appropriate instrument? Rheumatology (Oxford) 1999; 38: 878–882. 10515650 10.1093/rheumatology/38.9.878

[19] Chen YH , Huang WN , Chen YM , Lai KL , Hsieh TY , Hung WT , et al. The BASDAI cut-off for disease activity corresponding to the ASDAS scores in a Taiwanese cohort of ankylosing spondylitis. Front Med (Lausanne) 2022; 9: 856654. 35652077 10.3389/fmed.2022.856654 PMC9149077

[20] van der Heijde D , Deodhar A , Fleischmann R , Mease PJ , Rudwaleit M , Nurminen T , et al. Early disease activity or clinical response as predictors of long-term outcomes with certolizumab pegol in axial spondyloarthritis or psoriatic arthritis. Arthritis Care Res (Hoboken) 2017; 69: 1030–1039. 27696727 10.1002/acr.23092 PMC5518306

[21] Calin A , Garrett S , Whitelock H , Kennedy LG , O’Hea J , Mallorie P , et al. A new approach to defining functional ability in ankylosing spondylitis: the development of the Bath Ankylosing Spondylitis Functional Index. J Rheumatol 1994; 21: 2281–2285. 7699629

[22] Doward LC , Spoorenberg A , Cook SA , Whalley D , Helliwell PS , Kay LJ , et al. Development of the ASQoL: a quality of life instrument specific to ankylosing spondylitis. Ann Rheum Dis 2003; 62: 20–26. 12480664 10.1136/ard.62.1.20 PMC1754293

[23] Jenkinson TR , Mallorie PA , Whitelock HC , Kennedy LG , Garrett SL , Calin A. Defining spinal mobility in ankylosing spondylitis (AS). The Bath AS Metrology Index. J Rheumatol 1994; 21: 1694–1698. 7799351

[24] Duruöz MT , Doward L , Turan Y , Cerrahoglu L , Yurtkuran M , Calis M , et al. Translation and validation of the Turkish version of the Ankylosing Spondylitis Quality of Life (ASQOL) questionnaire. Rheumatol international. 2013;33:2717–22. 10.1007/s00296-013-2796-y 23765201

[25] Ozer HTE , Sarpel T , Gulek B , Alparslan ZN , Erken E. The Turkish version of the Bath Ankylosing Spondylitis Functional Index: reliability and validity. Clinical Rheumatology. 2005;24:123–8. 15340866 10.1007/s10067-004-0984-6

[26] Akkoc Y , Karatepe AG , Akar S , Kirazli Y , Akkoc N. A Turkish version of the Bath Ankylosing Spondylitis Disease Activity Index: reliability and validity. Rheumatol Int 2005; 25: 280–284. 14730386 10.1007/s00296-003-0432-y

[27] Erden Y , Coskun M , Sertdemir A , Ugurlu H. Effects of cardiac rehabilitation on cardiac autonomic functions in ankylosing spondylitis patients: A randomized controlled study. Ann Med Res 2021; 28: 1103–1110.

[28] Surucu GD , Yildirim A , Yetisgin A , Akturk E. Epicardial adipose tissue thickness as a new risk factor for atherosclerosis in patients with ankylosing spondylitis. J Back Musculoskelet Rehabil 2019; 32: 237–243. 30248026 10.3233/BMR-160650

[29] Kitas GD , Gabriel SE. Cardiovascular disease in rheumatoid arthritis: state of the art and future perspectives. Annals of the rheumatic diseases. 2011;70:8–14. 21109513 10.1136/ard.2010.142133

[30] Gastaldelli A , Basta G. Ectopic fat and cardiovascular disease: what is the link? Nutr Metab Cardiovasc 2010; 20: 481–490. 10.1016/j.numecd.2010.05.005 20659791

[31] Marchington JM , Mattacks CA , Pond CM. Adipose tissue in the mammalian heart and pericardium: structure, foetal development and biochemical properties. Comp Biochem Physiol B 1989; 94: 225–232. 2591189 10.1016/0305-0491(89)90337-4

[32] Mazurek T , Zhang L , Zalewski A , Mannion JD , Diehl JT , Arafat H , et al. Human epicardial adipose tissue is a source of inflammatory mediators. Circulation 2003; 108: 2460–2466. 14581396 10.1161/01.CIR.0000099542.57313.C5

[33] Iacobellis G , Pistilli D , Gucciardo M , Leonetti F , Miraldi F , Brancaccio G , et al. Adiponectin expression in human epicardial adipose tissue in vivo is lower in patients with coronary artery disease. Cytokine 2005; 29: 251–255. 15749025 10.1016/j.cyto.2004.11.002

[34] Lambrecht S , Coudenys J , Keyser F , Verbruggen G , Deforce D , Elewaut D. Reduced levels of the TGFb family member GDF15 in spondyloarthritis versus other rheumatic diseases. Ann Rheum Dis 2011; 70: A1–A94.21381260

[35] Robertson LP , Davis MJ. A longitudinal study of disease activity and functional status in a hospital cohort of patients with ankylosing spondylitis. Rheumatology (Oxford) 2004; 43: 1565–1568. 15353608 10.1093/rheumatology/keh386

